# The pressure/volume relationship during dobutamine stress echocardiography in transplanted heart: comparison with quality of life and coronary anatomy

**DOI:** 10.1186/1476-7120-10-44

**Published:** 2012-11-14

**Authors:** Giovanni Minardi, Giordano Zampi, Amedeo Pergolini, Giovanni Pulignano, Massimiliano Scappaticci, Francesca Moschella Orsini, Gaetano Pero, Paola Lilla Della Monica, Giovanni Cioffi, Francesco Musumeci

**Affiliations:** 1 Department of Cardiovascular Science, Heart Transplant Center, “S. Camillo-Forlanini” Hospital, Circonvallazione Gianicolense, 87, Rome, 00151, Italy; 2 Department of Cardiovascular Science, Catheterization Laboratory, “S. Camillo-Forlanini” Hospital, Rome, Italy; 3 Department of Cardiology, Villa Bianca Hospital, Trento, Italy

**Keywords:** Dobutamine stress echocardiography, Cardiac allograft vasculopathy, Coronary angiography, Multislice computed tomography

## Abstract

**Background:**

Cardiac allograft vasculopathy (CAV) is a major late complication in cardiac transplant recipients and has a relevant impact on outcome of these patients. Aims of this study: to compare, in cardiac transplant recipients patients, the diagnostic value of pressure/volume relationship (ESPVR) during dobutamine stress echocardiography (DSE) for coronary artery disease, assessed by Multislice Computed Tomography (MSCT), and by coronary angiography (CA). We also analyzed any possible relationship between ESPVR and the Health Related Quality of Life of the patients (HRQoL), evaluated by SF–36 questionnaire.

**Methods:**

25 consecutive patients underwent DSE within 24 hours after MSCT coronary angiogram and then they underwent CA. The HRQoL questionnaire was administered to the patients in the settings of DSE. They were followed-up for 6 months.

**Results:**

DSE has a sensitivity in detecting CAV of 67%, specificity of 95%, positive predictive value of 67% and negative predictive value of 95%; DSE with ESPVR has a sensitivity of 100%, specificity of 95%, positive predictive value of 75%, negative predictive value of 100%; MSCT has a sensitivity of 100%; specificity of 82%; positive predictive value of 43%; negative predictive value of 100%. Htx recipients with a flat-biphasic ESPVR, although asymptomatic, perceived a worst HRQoL compared with the up-sloping ESPVR population, and this is statistically significant for the general health (p 0.0004), the vitality (p 0.0013) and the mental health (p 0.021) SF-36 subscale.

**Conclusions:**

Evaluation with DSE and ESPVR is accurate in the clinical control of heart transplant recipients reserving invasive evaluation only for patients with abnormal contractility indexes.

## Background

Cardiac allograft vasculopathy (CAV) is a major late complication in cardiac transplant recipients. This disease is insidious and usually gives no clues until heart failure, myocardial infarction, or death occurs [1-4]. Routine evaluation with coronary angiography and/or intravascular ultrasound are not a reasonable approach because invasive and potentially harmful, expensive, and sometimes inaccurate. Less invasive methods in identifying patients at increased risk of CAV haves been tested in recent studies [5-8]; Multislice Computed Tomography (MSCT) and dobutamine stress echocardiography (DSE) have shown to be accurate diagnostic techniques for this harmful condition [9,10].

In recent years, Bombardini and co-workers [11-13] demonstrated that the left ventricular (LV) end-systolic pressure-volume relationship (ESPVR) was a prognostically useful noninvasive index of global contractility, associated with adverse outcome in unselected series of patients with negative exercise [11] and DSE [13].

The primary aims of this study was to assess, in cardiac transplant recipients patients, the accuracy of ESPVR (stated as index of LV myocardial contractility) in detecting CAV searched by MSCT and coronary angiography. We also analyzed any possible relationship between ESPVR and quality of life in these patients.

## Methods

### Study patients

From September 2011 to January 2012, asymptomatic heart transplanted recipients from the Heart Transplant Center of San Camillo - Forlanini Hospital in Rome were consecutively enrolled.

Study population comprised 25 patients who underwent DSE within 24 hours after MSCT coronary angiogram and, if the results of almost one of the two non-invasive diagnostic technique was positive, the patient underwent coronary angiography within 1 week after the tests, and if negative within 2 months. All patients were in sinus rhythm and on optimal and maximal tolerated pharmacological therapy, according to current guidelines for treatment and management of cardiac transplant [14]. Ongoing medical therapy was kept unchanged at the time of the stress test. No patients had implantable cardioverter-defibrillators or pacemakers. The exclusion criteria were: less than 12 months since transplant, iodinated contrast media allergy, signs or symptoms of CAV and New York Heart Association (NYHA) functional class IV. The sonographers were unaware of the results of the MSCT and of the coronary angiography.

The study complies with the Declaration of Helsinki. All patients gave written informed consent when they underwent stress echocardiography. When patients signed the consent forms, they also authorized physicians to use their clinical data according to Italian law.

### Quality of life assessment

Quality of life has been assessed with the standard form of the SF–36 Health Survey (Version 1) [15], which has been used in a number of previous studies of organ-transplant candidates and recipients [16], with findings suggesting excellent validity and reliability. This test includes multiple-item scales to measure the following 8 health-related aspects: physical function (PF); role-physical (RP); bodily pain (BP); general health perceptions (GH); vitality (VT); social function (SF); role-emotional (RE); and mental health (MH). Scores were transformed into scores from 0 to 100, based on SF–36 scoring algorithms, where higher scores reflect better health [17]. SF-36 questionnaire was administered to the patients in the settings of DSE.

### Stress protocol

Two-dimensional echocardiography and 12-lead electrocardiographic monitoring were performed in combination with high dose (up to 40 mcg/kg/min) dobutamine. Atropine infusion was considered if 85% of maximal heart rate wasn’t achieved at the end of DSE. During the procedure, blood pressure and the electrocardiogram were recorded each minute. Only representative cycles with optimal endocardial visualization were measured and the average of three measurements was taken. Images were acquired at baseline and at each 10-beat frequency increase during stress. LV volumes and ejection fraction were measured at rest and at each step using biplane Simpson rule. All volume measures were normalized by dividing by body surface area. Regional wall motion was assessed according to the recommendations of the European Society of Echocardiography from 1 (normal) to 4 (dyskinetic) in a 16-segment model of the left ventricle [18]. The blood pressure recording was made using a sphygmomanometer and the diaphragm of a standard stethoscope [19].

Non-echocardiographic criteria for ending the test were peak dobutamine, achievement 85% of target heart rate, (determined according to the equation: 220 – age), intolerable chest pain, excessive increase in blood pressure (defined as systolic blood pressure > 220 mm Hg; diastolic blood pressure > 120 mm Hg), hypotension (relative or absolute: > 30 mm Hg decrease in blood pressure), sustained supraventricular arrhythmias (supraventricular tachycardia or atrial fibrillation), ventricular arrhythmias (ventricular tachicardia, frequent, polymorphous premature ventricular beats), bradyarrhythmias. A maximal test was defined by the achievement of 85% of age-predicted maximal heart rate.

### End-systolic pressure–volume determination

ESPVR was defined as the ratio of the systolic blood pressure (SBP)/end-systolic volume index (ESVi). The slope of the relationship was calculated as the ratio between SBP/ESVi and heart rate increase (from baseline to peak stress). The ESPVR was defined up-sloping when dobutamine-induced SBP/ESVi increase was higher than 25^th^ percentile values of the entire study group, flat when below the 25^th^ percentile values; biphasic, with an initial up-sloping followed by a later down-sloping trend, when peak dobutamine SBP/ESVi was lower than intermediate stress values [20,21]. The critical heart rate was defined as the heart rate at which SBP/ESVi reached the maximum value during progressive increase in heart rate. In biphasic pattern, the critical heart rate was the heart rate beyond which SBP/ESVi declined by 5%.

### Clinical follow-up

The patients were observed prospectively for 6 months with regular monthly outpatient visits. Adverse cardiovascular events were registered. The events considered were death, non-fatal acute myocardial infarction, and heart failure.

### Statistics

Statistical analyses were performed using SPSS, version 17.0 (SPSS, Inc., Chicago, IL) for Windows. We assumed the coronary angiography as the reference standard for the identification of CAV. Results of MSCT and DSE (with and without ESPVR) were compared to the coronary angiography’s ones in order to evaluate diagnostic accuracy of the non-invasive tests. Distribution of relevant variables was summarized using frequencies, means, medians and standard errors (SEs), as appropriate. Univariate analysis was performed to compare the differences between ESPVR with continuous variables, using Fisher’s exact test and the Student unpaired *t*-test. Differences between groups and quality of life measures were analyzed using tests for non-normally distributed samples, as appropriate. To address the potential problem of error rate with multiple testing, Bonferroni–Holm adjustments were performed. All probability values were obtained using 2-sided analyses; p ≤ 0.05 was considered statistically significant for all analyses.

## Results

The baseline clinical and echocardiographic characteristics of the study patients are reported in Table 1. Most of patients were males in NYHA functional class I-II with a high prevalence of history of hypertension. Resting LV ejection fraction was normal in all patients. About three fourth of patients received beta-blockers and 23 of 25 took statins and acetyl salicylic acid. In the study protocol 3 patients with percutaneous transluminal coronary angioplasty (PTCA) procedure with stent deployment after HTx were enrolled: 2 of them underwent procedure, about 5 years before, because of a ST-elevation-Myocardial Infarction (door-to-balloon < 90 min) without any residual stenosis nor myocardial damages, and 1 of them due to a significant coronary stenosis founded 7 years before during a follow-up coronary angiography.

**Table 1:** Characteristics of the study patients

	
Patients	25
Age (years)	58 ± 12
Gender (M/F)	21/4 (84/16%)
Years from Htx	8.5 ± 7
BMI (kg/m^2^)	27.4 ± 4.2
eGFR - CKD-EPI (ml/min)	61.7 ± 25.8
Hypertension	19 (76%)
Diabetes	10 (40%)
Previous Smoking Habits	17 (68%)
Metabolic Syndrome	8 (32%)
Familiarity for CVD	12 (48%)
Dyslipidemia	12 (48%)
Atypical Chest Pain	6 (24%)
PTCA (post HTx)	3 (12%)
Myocardial Infarction (post HTx)	2 (8%)
NYHA class (I-IV)	
I	11 (44%)
II	12 (48%)
III	2 (8%)
IV	
**Cause of Htx**	
Post Ischemic Dilated CM	9 (36%)
Idiopathic Dilated CM	11 (44%)
End-Stage Hypertrophic CM	1 (4%)
Non-Compactation CM	1 (4%)
Peripartum CM	1 (4%)
Dilated CM in Becker Dystrophy	1 (4%)
Post Myocarditis Dilated CM	1 (4%)

*BMI*, Body mass index; *eGFR*, Estimated Glomerular Filtration Rate; *CKD*-*EPI*, Chronic Kidney Disease Epidemiology Collaboration; *CVD*, cardiovascular disease; *Htx*, heart transplantation; *NYHA*, New York Heart Association; *CM*, Cardiomyopathy.

### Feasibility and tolerability of high-dose DSE

Near the totality of patients (22/25 = 88%) reached the maximal dose of DSE (40 mcg/kg/min); in 3 patients dobutamine infusion was stopped at 30 mcg: in 1 patient because of the achievement of 85% of age-predicted maximal heart rate, and in 2 patients because of excessive increase in blood pressure.

5 patients (20%) achieved age-predicted maximal heart rate at the end of DSE, whilst the remaining 20 patients achieved 85% of maximum predicted heart rate. During dobutamine infusion, non-sustained ventricular tachycardia was observed in 1 patient; this complication was well tolerated, not causing the premature interruption of the test. Hemodynamic and echocardiographic responses to DSE are described in Table 2.

**Table 2:** **Echocardiographic data and force**-**frequency relationship during dobutamine echo**

	
Left Ventricular EDV (ml)	108 ± 28
Left Ventricular ESV rest (ml/m^2^)	23 ± 8
Left Ventricular ESV peak (ml/m^2^)	11 ± 6
Systolic Pressure rest (mmHg)	127 ± 13
Systolic Pressure peak (mmHg)	160 ± 27
SP/ESV index (rest)	6.22 ± 2.53
SP/ESV index (peak)	17.03 ± 7.88
ESPVR slope (χ 10^-2^)	21.1 ± 7.3
Δ peak – rest (mmHg/ml)	10.85 ± 6.9
Heart rate rest (b.p.m.)	82 ± 6
Heart rate peak (b.p.m.)	129 ± 15
Left Ventricular Ejection fraction (rest, %)	59 ± 8
Left Ventricular Ejection fraction (peak, %)	72 ± 9
Double product (rest)	10437 ± 1396
Double product (peak)	20838 ± 3481
Ischaemia during Dobutamine, n (%)	3 (12%)
Flat-biphasic ESPVR during Dobutamine, n (%)	4 (16%)
Critical Heart Rate (b.p.m.)	123 ± 17
Dobutamine dose 40 μg, n (%)	22 (88%)
Dobutamine dose 30 μg, n (%)	3 (12%)
**On therapy at the time of stress**:	
Everolimus	10 (40%)
Tacrolimus	5 (20%)
Cyclosporine	22 (88%)
Mycophenolate mofetil	14 (56%)
Cortisone	3 (12%)
Ca-Channel Blocker	6 (24%)
Nitrates	3 (12%)
B-Blocker	19 (76%)
Diuretics	12 (48%)
Acetyl salicylic acid	23 (92%)
Amiodarone	1 (4%)
ACE-I	13 (52%)
ARBs	2 (8%)
Statins	23 (92%)

*ACE*-*I*: angiotensin converting enzyme inhibitor; *ARBS*: Angiotensin II Receptor Blockers; *EDV*: end diastolic volume; *ESPVR*: end-systolic pressure/volume ratio; *ESV*: end systolic volume; *SP*, systolic pressure.

### Relation among DSE, DSE + ESPVR, MSCT and CA

At the coronary angiography 3 out of 25 patients (12%) had significant coronary stenosis: a 80% narrowed proximal left anterior descending artery was found in all the 3 patients.

The DSE resulted positive in 2 patients with positive coronary angiography and in 1 out of 25 patients with negative coronary angiography; 1 patient with CAV had a negative test (sensitivity 67%; specificity 95%; positive predictive value 67%; negative predictive value 95%).

**Figure 1:**
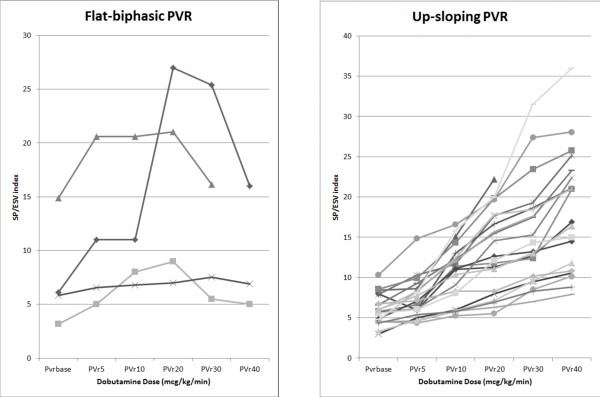
Plot of the force–frequency relationship in Group 1 (flat-biphasic ESPVR, left panel) and Group 2 (up-sloping-ESPVR, right panel).

The ESPVR was abnormal (flat or biphasic) in 4 patients and normal (i.e. steep up-sloping) in 21 patients. The biphasic response was present in all the patients with positive coronary angiography (3/3) and in 1 out of 25 patients with negative coronary angiography (sensitivity 100%; specificity 95%; positive predictive value 75%; negative predictive value 100%) (Figure 1).

The MSCT showed significant coronary artery stenosis in 7 out of 25 patients: all the 3 patients with CAV were correctly identified (showing the same results of the coronary angiography), but 4 patients with positive MSCT had normal coronary arteries at the coronary angiography (sensitivity 100%; specificity 82%; positive predictive value 43%; negative predictive value 100%).

### Comparison of quality of life ratings with normative data

**Figure 2:**
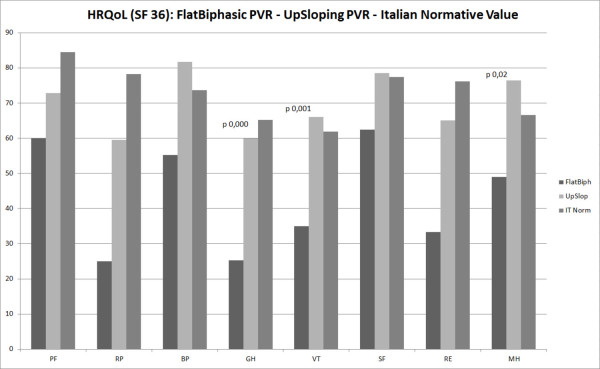
Comparison of quality of life (SF 36) between heart transplanted patient with Flat-biphasic ESPVR, up-sloping ESPVR and age-adjusted Italian population Normative value (PF: physical function; RP: role-physical; BP: bodily pain; GH: general health perceptions; VT: vitality; SF: social function; RE: role-emotional; MH: and mental health).

Patients’ quality of life self-ratings were compared with age- and gender-adjusted norms [17]. Based on normative data, HTx recipients with up-sloping ESPVR perceived a better quality of life for 4 of 8 SF-36 subscales compared with the normative population. Htx recipients with a flat-biphasic ESPVR, perceived a worset quality of life compared with the up-sloping ESPVR population, and this was statistically significant for the GH (Flat-biphasic 25.25 ± 19.2 vs up-sloping 60 ± 15, p 0.0004), the VT (Flat-biphasic 35 ± 9 vs up-sloping 66 ± 16, p 0.0013) and the MH (Flat-biphasic 49 ± 24.7 vs up-sloping 76.4 ± 19.6, p 0.021) SF-36 subscale (Figure 2).

Quality of life was independent of principal demographic variables such as age, gender and years from transplantation (Table 3).

**Table 3:** **Statistics correlations between demographic and echocardiographic characteristics of the study population and the SF**-**36 items**

	**PF**	**RP**	**BP**	**GH**	**VT**	**SF**	**RE**	**MH**
Demographics								
Age (years)	*p* 0.27	*p* 0.09	*p* 0.7	*p* 0.13	*p* 0.20	*p* 0.73	*p* 0.12	*p* 0.89
Gender (M/F)	*p* 0.06	*p* 0.19	*p* 0.12	*p* 0.46	*p* 0.25	*p* 0.22	*p* 0.49	*p* 0.21
TimeHTx	*p* 0.22	*p* 0.29	*p* 0.92	*p* 0.78	*p* 0.45	*p* 0.24	*p* 0.19	*p* 0.92
Echocardiographic variable								
Flat-bifasic	*p* 0.27	*p* 0.16	*p* 0.67	*p* 0.000^	*p* 0.001^	*p* 0.22	*p* 0.21	*p* 0.02*

*TimeHTx*, time from heart transplant; * *p* value < 0.05; ^ *p* value < 0.01.

### Results of 6 months follow-up

At the end of the follow-up no patient died; during the 6 months of follow-up, 2 patients, both with positive DSE and flat-biphasic ESPVR, had cardiac events.

1 patient, with coronary stenosis treated with stenting, experienced, two months later, an exacerbation of chronic heart failure solved ambulatory by increasing diuretics doses.

1 patient, with negative MSCT and CA but positive DSE and flat-biphasic ESPVR, experienced, four months later, an acute heart failure episode due to acute heart rejection diagnosed by endo-myocardial biopsy. The patient was hospitalized and treated with a short course of high-dose corticosteroids, a calcineurin inhibitor and an anti-proliferative agent; thanks to this treatment, the patient survived to the episode.

## Discussion

Differently from general population of patients with coronary artery disease, there are only few studies comparing the accuracy of DSE, MSCT and coronary angiography for detecting CAV in cardiac transplant recipients [10]. The uniqueness of our study is that all our patients underwent the three techniques, and introduced in the DSE protocol the valuation of the ESPVR for diagnosing CAV. At our knowledge, indeed, this is the first study in which ESPVR is used to assess LV contractility, coronary anatomy and quality of life in a population of asymptomatic heart transplant recipients. The results of the present study are broadly consistent with the previous evidences suggesting that the challenge of inotropic reserve with catecholamine infusion is useful to unmask depressed contractile reserve in a left ventricle with latent dysfunction. Even more clinically relevant and original, this technique significantly improves the prediction of CAV in comparison with MSTC, reducing the risk of useless invasive interventions.

Difficulty in detecting and treating CAV remains the major limiting factor for survival after heart transplantation. The disease is silent in most of cases mainly because of cardiac denervation.

First modality and diagnostic test for detecting CAV is the anatomic one: coronary angiography CA is still the reference standard for diagnosing CAV, in spite of its invasive nature not free from complications and the exposure to ionizing radiation and to iodinated contrast media given to patients taking immunosuppressive medications. Up today, many centers perform annual evaluations to establish the presence and severity of CAV after heart transplantation [22].

Another relevant anatomic modality to detecting CAV, although non-invasive, is MSCT. In 2000, Knollmann and co-workers [20] compared, in 112 heart transplant recipients, the electron-beam CT features of coronary arteries with those of biplane coronary angiography and intracoronary ultrasound. They found that electron-beam CT had a sensitivity of 94%, a specificity of 79%, a positive predictive value of 43%, and a negative predictive value of 99% for detecting coronary stenosis. Similar results, comparing cardiac multi-detector computed tomography angiography and coronary angiography, were found by von Ziegler and co-workers [21]. From these data appear a high negative predictive value and an unsatisfactory positive predictive value of this diagnostic tool. Our results are in line with those previously reported that lead to overestimate the coronary disease with the clinical implication of an inappropriate request for coronary angiography, which implies the use of nephrotoxic agents. As expected, many heart transplant recipients have chronic kidney disease due to cyclosporine or other immunosuppressive therapy: in our population 88% of the patients were treated with cyclosporine. It is a very important problem that the cardiologists have to think about when prescribe a MSCT.

Moreover, we analyzed the presence of any possible relationship between ESPVR and quality of life. Theoretically a loss of contractile reserve could carry to a worse physical activity: it can be caused by a classic ischemic mechanism, due to a reduced blood supply in situations of increased cardiac output (i.e. catecholamine infusion), or by an inflammatory response as in the heart transplant rejection. Both mechanisms cannot be identified either by CA or by MSCT, and a functional test is requested to reveal whether a reduced functional capacity may be associated with a latent ventricle dysfunction.

Our results seem confirm this hypothesis: patients with flat-biphasic ESPVR, indeed, have lower scores in all the 8 subscales of the SF-36 questionnaire, and this is statistically significant for general health, mental health and vitality. Also in that single patient with flat-biphasic ESPVR and negative coronary angiography, the results of the SF-36 were coherent with the inotropic reserve found.

### Study limitations

Our study has several limitations.

Calculation of the ESPVR requires measurement of the LV pressure at the end-systole. Because only non-invasive measurements were available, cuff systolic pressure was substituted for end-systolic pressure.

The overall patient population was small, thus limiting the generalizability of our findings. Studies with a larger sample size and longer follow-up are warranted.

Finally, in all patients on β-blocking agents, therapy was kept unchanged and may have influenced, at least in part the results. However, the optimal dose of dobutamine to assess contractility and recognize CAV is not defined and it did not appear ethical to withdraw a life-saving therapy.

## Conclusions

The evaluation of ESPVR with DSE may be a safe and feasible strategy for the clinical control of heart transplant recipients during the time. It seems to provide an accurate assessment of coronary artery anatomy and function and a suitable selection of candidates to invasive evaluation. ESPVR evaluation may also be helpful for explaining a worsen quality of life referred by some patient when it is associated with the development of cardiac abnormalities during physical exercise undetectable by most of the available invasive and non-invasive diagnostic techniques for CAV.

## Competing interests

The authors declare that they have no competing interests.

## Authors’ contributions

GM: concept and design, analysis and interpretetion, critical revisions and data collection; GZ: concept and design, analysis and interpretetion, drafting manuscript and data collection; AP: analysis and interpretetion and data collection; GP: concept and design, analysis and interpretetion and critical revisions; MS: literature search and data collection; FMO: literature search and data collection; GP: literature search and data collection PLDM: literature search and data collection. GC: analysis and interpretetion, drafting manuscript and data collection; FM: analysis and interpretation and critical revisions. All authors read and approved the final manuscript.
